# 877 Evaluating First Positive Cultures in Burns: Rethinking Broad-Spectrum Antibiotic Choices

**DOI:** 10.1093/jbcr/iraf019.408

**Published:** 2025-04-01

**Authors:** Pouriya Sadeghighazichaki, Alan Rogers, Philip Lam, Stephanie Mason, Marion Elligsen, Rimona Natanson, David Wallace

**Affiliations:** Ross Tilley Burn Centre, Sunnybrook Health Sciences Centre; Ross Tilley Burn Centre, Sunnybrook Health Sciences Centre; Division of Infectious Diseases, Sunnybrook Health Sciences Centre; Ross Tilley Burn Centre, Sunnybrook Health Sciences Centre; Sunnybrook Health Sciences Centre; Ross Tilley Burn Centre, Sunnybrook Health Sciences Centre; Ross Tilley Burn Centre, Sunnybrook Health Sciences Centre

## Abstract

**Introduction:**

Nearly all major burn patients experience an infection during their admission. Broad-spectrum antibiotics both alone and in combination may have adverse effects and encourage multi-drug resistance. The purpose of this study is to determine the most appropriate antibiotics for major burn patients on first positive culture (FPC).

**Methods:**

This was a retrospective study of adult burn patients with >20% TBSA at an ABA verified burn centre from 2018 to 2023. Patients were included if they had at least one positive culture from blood, sputum, urine or wound during their admission or received antibiotics without positive cultures. Standard clinical information was collected along with the first organism cultured and antibiotic used. The primary outcome was the bacterial profile on first positive culture, stratified by sterile (blood and OR culture) and non-sterile (sputum, wound, and urine), and by early (< 5 days) and late (>5 days). The secondary outcome was the empiric antibiotic prescribed on FPC taken. The tertiary outcome was the need for Vancomycin on FPC based on admission MRSA screening results. Descriptive analysis was completed.

**Results:**

The study population included 144 patients. Median age was 47 (36-59 IQR), 106 were male (74%), median TBSA was 32%, 31 (22%) had inhalation injury and 102 (71%) were mechanically ventilated. One-hundred-nineteen (83%) patients had at least one positive culture: respiratory (58%), wound (27%), blood (8%), and urine (7%). Of all FPCs, most were non-sterile (83%) and early (61%). Most common FPC organisms grown early were MSSA (26%), Haemophilus influenzae (21%), Enterococcus species (9%) and Escherichia coli (7%). Most common FPC organisms grown late were MSSA (17%), Pseudomonas species (12%), Escherichia coli (10%), Enterobacter cloacae complex (8%), and Enterococcus Species (8%). Of all patients included, 141 (98%) were started on antibiotics with the majority prescribed piperacillin/tazobactam (39%), cefazolin (23%), vancomycin (13%) and ceftriaxone (9%). Only 15 (10%) of patients were started on combination therapy. Finally, of the 115 patients screening negative on admission for MRSA, only 4 (3%) had positive MRSA cultures on FPC.

**Conclusions:**

The results from this study suggest that single agent antibiotics are sufficient for first positive culture. Given the low incidence of Pseudomonas isolated from patients early in admission, ceftriaxone appears to be a reasonable empirical choice of therapy when infection is suspected. There is little need for initiation of Vancomycin on FPC if admission MRSA screen is negative.

**Applicability of Research to Practice:**

This study helps guide antimicrobial use for the first infection experienced by major burn patients during their admission.

**Funding for the Study:**

N/A

